# Simple oncoplastic breast defect closure improves long-term cosmetic outcome of breast conserving surgery for breast cancer: A randomised controlled trial

**DOI:** 10.1016/j.breast.2022.07.001

**Published:** 2022-07-18

**Authors:** Christopher Hadjittofi, Hend Almalki, Bahar Mirshekar-Syahkal, Simon Pain, Katalin Zechmeister, Maged Hussien

**Affiliations:** Department of Breast Surgery, Norfolk and Norwich University Hospitals, Colney Lane, Norwich, NR4 7UY, United Kingdom

**Keywords:** Breast, Cancer, Oncoplastic, Cosmesis, Satisfaction

## Abstract

**Introduction:**

Breast conserving surgery (BCS) is associated with unsatisfactory cosmetic outcomes in up to 30% of patients, carrying psychological and quality-of-life implications. This study compares long-term cosmetic outcomes after BCS for breast cancer with v without simple oncoplastic defect closure.

**Methods:**

A randomised controlled trial was performed, recruiting patients who underwent BCS over four years and randomising to the “reshaping” group (closure of excision defect with mobilised breast tissue; n = 124) and to the “control” group (no attempt at defect closure; n = 109). The estimated excision volume (EEV) was <20% of breast volume (BV) in both groups. Photography and breast retraction assessment (BRA) were recorded preoperatively. Cosmetic outcomes were blindly assessed annually for five years by BRA, panel assessment of patients, and body image questionnaire (BIQ).

**Results:**

There were no significant differences between the reshaping and control groups in mean age (52.4 v 53.0; p = 0.63), body mass index (27.8 v 27.7; p = 0.80), margin re-excision (9 v 9; p = 0.78), mean BV (562.5 v 590.3 cc; p = 0.56), mean EEV (54.6 v 60.1 cc; p = 0.14), mean EEV/BV ratio (11.2 v 11.0; p = 0.84), or mean specimen weight (52.1 v 57.7 g; p = 0.24). Reshaping group patients had significantly better outcomes compared to control group patients in terms of mean BRA (0.9 v 2.8; p < 0.0001), achieving a score of “good” or “excellent” by panel assessment at 5 years (75.8% v 48%, p < 0.0001), body image questionnaire top score at 5 years (66.9% v 35.8%; p = 0.0001).

**Conclusions:**

Simple oncoplastic closure of defects after breast-conserving surgery improves long-term objective and subjective cosmetic outcomes.

## Highlights

• Breast-conserving cancer surgery can lead to unsatisfactory cosmetic outcomes.
• Simple oncoplastic closure improves long-term cosmetic outcomes.
• Objective and subjective cosmetic outcomes of oncoplastic closure are correlated.

## Introduction

Breast-conserving surgery (BCS) has overtaken mastectomy, becoming the standard of care for breast cancer [1]. Approximately 50–70% of women with breast cancer are currently treated with BCS [2]. Since BCS is associated with equivalent disease-free and overall survival to mastectomy [[3], [4], [5]] the focus of outcome assessment has shifted to cosmetic appearance, patient satisfaction, and quality of life [3].

Although BCS is the standard of care, unfortunately 30–40% of patients report disappointment with cosmetic results [2]. Poor cosmesis is usually characterised by breast or nipple asymmetry and/or contour defects [2]. Several risk factors of poor cosmetic outcomes have been described, including small breast, large tumour size, excision volume (i.e. excision-to-breast volume ratio of >20%), tumour location (i.e. medial tumours), postoperative complications (e.g. seroma or infection), and radiotherapy-related parameters (boost volume, photons, dose and beam orientation) [2,6,7].

Breast asymmetry following BCS is associated with fear of recurrence and impaired quality of life (particularly psychosocial functioning) [2]. Women with marked breast asymmetry are more likely to experience stigma and depression, and less likely to report improvements in their health after BCS [8]. Good cosmetic outcomes after BCS are therefore essential to psychological and global wellbeing.

Oncoplastic BCS offers reconstructive options to optimise cosmesis. Oncoplastic techniques can be broadly categorised into either “volume displacement” or “volume replacement”. In BCS, volume displacement techniques are most frequently used, and are graded according to defect volume/breast volume ratio (i.e. Level I: <10% of breast volume; Level II >10% of breast volume) [2]. In a recent systematic review of 78 non-randomised studies [9], oncoplastic defect closure was suggested as possibly superior to standard BCS in cosmetic outcomes, but the evidence base was characterised as “poor” [9].

### Aim of study

To compare long-term cosmetic outcomes of simple oncoplastic closure of breast defect v standard BCS for breast cancer.

## Methods

### Study design

This randomised controlled trial was approved by the East of England – Norfolk Research Ethics Committee (Reference No.: 06/Q0101/34), was registered with the Australian New Zealand Clinical Trials Registry (Registration No.: ACTRN12612000638831) [10] and carried out according to the Declaration of Helsinki. All patients were given information leaflets and provided written consent for trial participation.

Between April 2008 and August 2012, patients with early primary breast cancer who were due to undergo BCS at the Norfolk & Norwich University Hospital, were recruited. The inclusion criteria were: female patients over the age of 18 with primary breast cancer, where BCS (wide local excision) was indicated. The exclusion criteria were: Patients with an estimated-excision-volume-to-breast-volume (EEV/BV) ≥20%, or who required any oncoplastic procedures beyond simple closure/volume displacement.

Patients were randomised to either the “reshaping” (i.e. BCS with simple oncoplastic closure) or to the “control” (i.e. BCS alone) group by a computer-generated randomisation list and a sealed-envelope technique, facilitated by the University of East Anglia (UEA) Statistics Department.

### Baseline measurements

In addition to documentation of patient age and BMI, pre-treatment breast photography was performed, as well as baseline breast retraction assessment (BRA), breast volume assessment, and estimation of excision volume. All measurements were carried out by the same breast surgeon.

BRA was carried out as previously described [11,12] and according to the following formula:

BRA = √((X_R_ – X_L_)^2^ + (Y_R_-Y_L_)^2^)
X_R_ = distance from midline to right nipple (cm)
X_L_ = distance from midline to left nipple (cm)
Y_R_ = distance from right midclavicular point to right nipple (cm)
Y_L_ = distance from left midclavicular point to left nipple (cm)

The breast volume (BV) was calculated by the Sloane formula [12]:

13πr2h

r = half the diameter of the breast base (i.e. distance between the inframammary fold and the upper border of the breast on a lateral mammogram) cmh = the height of the breast (i.e. the distance from the nipple to the pectoralis major on a lateral mammogram at 90° angle) cm

π=3.141

The EEV/BV ratio was calculated as follows:

4 (radius of tumour + 1 cm)^3^

(r)^2^ x height of breast

(Radius of tumour = maximum diameter of the tumour on mammogram in cm)

### Surgical technique

**Fig. 1:**
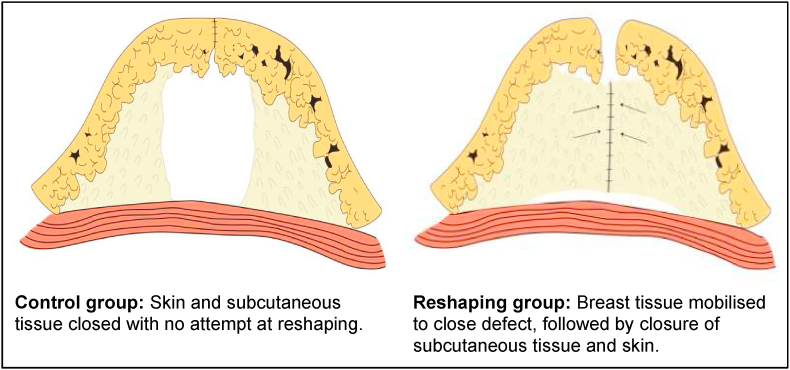
Surgical technique.

In the reshaping group, following wide local excision (WLE) of the breast tumour, the adjacent breast tissue was mobilised from the skin and pectoral muscles, and was displaced to fill the defect. Breast tissues from the defect margins were approximated using interrupted absorbable sutures (2-0 polyglactin 910; Vicryl©, Ethicon US, LLC), as was the deep dermis. Skin closure was performed with continuous subcuticular absorbable sutures (4-0 poliglecaprone 25; Monocryl©, Ethicon US, LLC). The incisions were infiltrated with Bupivacaine at a dose of <2.0 mg/kg. In the control group, there was no mobilisation or approximation of breast tissues. Local anaesthetic infiltration, deep dermal and skin closure were performed as described above (Fig. 1).

The operations were carried out by four surgeons and the resection specimen was weighed prior to formalin fixation. Patients were treated on a day-case basis according to standard local departmental clinical practice. Non-palpable tumours were localised using the ROLL (radioguided occult lesion localisation) technique. Sentinel lymph node biopsies using radioisotope technique and axillary dissections were performed according to local unit protocol and usually performed through a separate axillary incision.

All patients received radiotherapy 6–8 weeks following surgery. The standard regimen involved 40 Gy in 15 fractions within 3 weeks. Patients received adjuvant treatment according to local unit protocol, depending on postoperative histology.

### Outcome measures

In addition to their standard clinical follow-up, for the purposes of this study patients were postoperatively followed-up annually for five years. Cosmetic outcomes were assessed using objective methods (BRA), subjective methods (panel assessment), as well as a body image questionnaires. All patients had a photograph after each assessment in the hospital's Medical Illustration Department. All photographs were taken using the same camera and magnification. The photograph background was a standard blue, with a black horizontal line adjusted at the level of the suprasternal notch and a black vertical line adjusted at the midline.

BRA was performed as described above and recorded prospectively on the data collection forms. All measurements were performed by the same breast surgeon and recorded prospectively.

Panel assessments were performed by a breast surgeon and a nurse, who were blinded to the randomisation. Assessment was based on specific criteria, including breast shape, size, contour and symmetry. Radiotherapy changes, such as breast skin oedema, pigmentation or telangiectasia, were also assessed. Each parameter were scored independently by each assessor and recorded without discussion between the two assessors. An overall assessment score was given by each assessor independently and recorded prospectively. The panel graded the cosmetic outcomes along the following scale:

• Excellent (9–10): treated and untreated breast appear nearly identical
• Good (7–8): slight difference in appearance of breasts
• Fair (5–6): Clear difference but no major distortion in treated breast
• Poor (≤4): Major distortion in treated breast [13].

The body image questionnaire [14] was completed annually by the patient and consisted of the following ten questions:

1. Have you been feeling self conscious about your appearance?
2. Have you felt less physically attractive as a result of your disease or treatment?
3. Have you been dissatisfied with your appearance when dressed?
4. Have you been feeling less feminine as a result of your disease or treatment?
5. Did you find it difficult to look at yourself naked?
6. Have you been feeling less sexually attractive as a result of your disease or treatment?
7. Did you avoid people because of the way you felt about your appearance?
8. Have you been feeling the treatment has left your body less whole?
9. Have you felt dissatisfied with your body?
10. Have you been dissatisfied with the appearance of your scar?

The response to each question was scored (“Not at all” = 1; “A little” = 2; “Quite a bit” = 3; “Very much” = 4), giving a best possible body image score (BIS) of 10 and a worst possible score of 40. The questionnaire was provided to patients by the breast clinic nurse immediately prior to clinical review by the breast surgeon. The patients completed the questionnaire in private and submitted it to the nurse for inclusion in their medical records. During follow-up visits, patients were not asked or influenced in any way to express how they think of their cosmetic outcome.

### Analysis

Statistical analysis was performed using IBM® SPSS v.23.

Parametric data are expressed as means with standard deviation (SD). Nonparametric are expressed as medians with interquartile range (IQR). Baseline variables and outcome measures were compared between patient groups Student's *t*-test or the Mann-Whitney *U* test as appropriate. Correlations were drawn with the Spearman rank test, and inter-observer agreement was assessed with Cohen's kappa. Statistical significance was set at α = 0.05 (p < 0.05) for all tests.

Power calculation: A sample size of 120 in each group was required, based on an anticipated 35% and 15% poor cosmetic result in the control and in the intervention group respectively, in order to achieve 90% power to detect a difference at the p = 0.05 level. To allow for a 20% drop-out, we planned to recruit 300 participants.

## Results

During the study period, 260 patients were approached for recruitment. After 14 patients declined to participate, preferring to receive standard treatment (WLE without reshaping), 246 were recruited and randomised to the control (n = 120) and reshaping (n = 126) groups. Eleven and two patients were lost to follow-up from the control and reshaping groups respectively, leaving 233 patients for analysis (control group, n = 109; reshaping group, n = 124).

There was no statistically significant difference (p > 0.05) between the reshaping and control groups in terms of mean age (52.4 v 53.0 years; p = 0.63), body mass index (27.8 v 27.7; p = 0.80), number of patients who underwent re-excision of margins (9 v 9; p = 0.78), mean breast volume (BV; 562.5 v 590.3 cc; p = 0.56), mean estimated excision volume (EEV; 54.6 v 60.1 cc; p = 0.14), mean EEV/BV ratio (11.2 v 11.0; p = 0.84), mean specimen weight (52.1 v 57.7 g; p = 0.24). In the majority of patients (127/233; 55%), the tumour was located in the upper outer breast quadrant. There was no statistically significant difference (p = 0.399) in tumour location between the reshaping and control groups, as presented in Table 1. Two patients (1.6%) in the reshaping group and one (0.9%) in the control group returned to theatre for drainage of haematoma (p = 1.00). Five patients (4.0%) in the reshaping group and 4 (3.7%) in the control group developed wound infections and subsequent fat necrosis at the wide local excision site (p = 1.00). All patients with would infections were treated with oral antibiotics and none required return to the operating theatre. The development of infection and fat necrosis did not influence the final cosmetic outcome.

**Table 1:** Tumour location by patient group and breast quadrant.

	Reshaping group (n = 124)	Control group (n = 109)	p-value^a^
**Lower inner quadrant**	23 (19%)	13 (12%)	0.399
**Lower outer quadrant**	22 (18%)	18 (17%)	
**Upper inner quadrant**	13 (10%)	17 (16%)	
**Upper outer quadrant**	66 (53%)	61 (56%)	

a p-value calculated by the chi-squared test, showing no statistically significant difference in the distribution of tumours between the reshaping and the control group.

Cosmetic outcomes were superior in the reshaping v the control group (p ≤ 0.0001), as quantified by breast retraction assessment, panel assessment, and body image score questionnaire score.

**Fig. 2:**
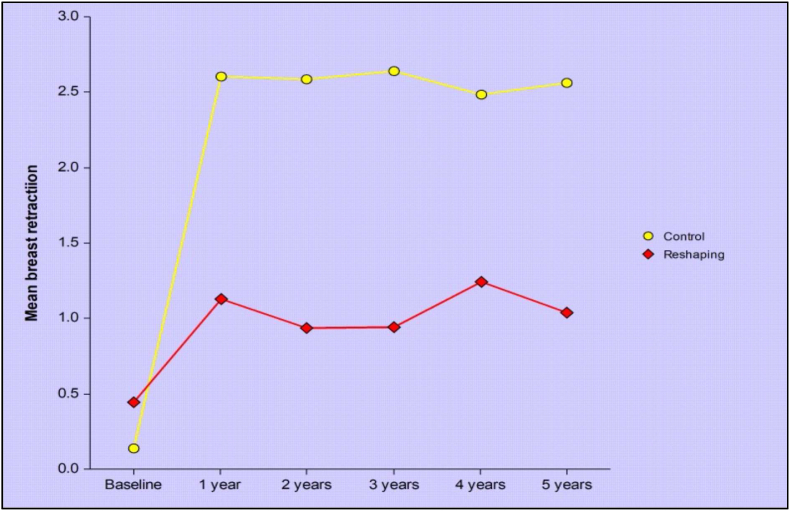
Mean breast retraction assessment values.

**Fig. 3:**
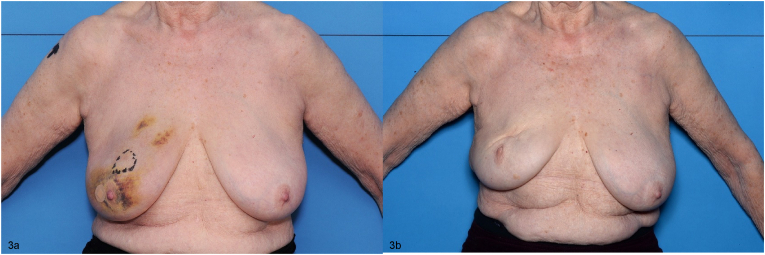
Preoperative (3a) and five-year postoperative (3b) photographs of control-group patient with tumour in the superior aspect of the right breast.

**Fig. 4:**
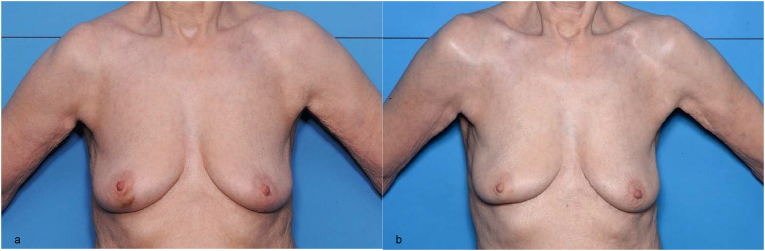
Preoperative (4a) and five-year postoperative (4b) photographs of reshaping-group patient with tumour inferior to the areola.

Mean BRA values were similar between the control and reshaping group at baseline (0.16 v 0.5), but were higher (i.e. poorer cosmetic outcome) in the control group v the reshaping group at each annual assessment (Year one: 2.8 v 1.2; Year two: 2.8 v 1.0; Year three: 2.7 v 1.0; Year four: 2.9 v 1.0; Year five: 2.8 v 0.9; p < 0.0001 for all) (Fig. 2). Photographic examples of the control and reshaping technique are presented in Fig. 3, Fig. 4 respectively.

Panel assessment significantly favoured the reshaping group at 5 years follow-up both by the breast surgeon and the nurse. Significantly more patients in the reshaping group v the control group received scores of either “good score 7–8” or “excellent score 9–10” (Table 2).

**Table 2:** Annual results of panel assessment and body image scores.

	Reshaping group (n = 124)	Control group (n = 109)	p-value^a^
**Year-one assessment:** **Good/excellent score by surgeon** **Good/excellent score by nurse** **Best BIQ score**^b^	94 (75.8%) 94 (75.8%) 40 (32%)	53 (48%) 55 (50.4%) 38 (35%)	< **0.0001** < **0.0001** 0.50
**Year-two assessment:** **Good/excellent score by surgeon** **Good/excellent score by nurse** **Best BIQ score**^b^	90 (75.5%) 92 (74%) 61 (49%)	50 (45.8%) 50 (45.8%) 41 (37.6%)	< **0.0001** < **0.0001** **0.03**
**Year-three assessment:** **Good/excellent score by surgeon** **Good/excellent score by nurse** **Best BIQ score**^b^	88 (71%) 88 (71%) 58 (46.7%)	43 (39.4%) 44 (40.3%) 40 (36.6%)	< **0.0001** < **0.0001** **0.07**
**Year-four assessment:** **Good/excellent score by surgeon** **Good/excellent score by nurse** **Best BIQ score**^b^	90 (72.5%) 89 (71.7%) 59 (47.5%)	46 (42.2%) 46 (42.2%) 39 (35.7%)	< **0.0001** < **0.0001** **0.06**
**Year-five assessment:** **Good/excellent score by surgeon** **Good/excellent score by nurse** **Best BIQ score**^b^	94 (75.8%) 94 (75.8%) 83 (66.9%)	49 (45%) 44 (40.3%) 39 (35.8%)	< **0.0001** < **0.0001** **0.0001**

a p-values calculated by the Mann-Whitney test.

b Best BIQ score: A score of 10/40 on the Body Image Questionnaire.

A significantly greater proportion of patients in the reshaping v the control group (66.9% vs. 35.8%; p = 0.0001) achieved the best BIS (i.e. a score of 10/40) at 5 years follow up (Table 2). The proportion of control-group patients achieving best BIS fluctuated between 35.0% and 37.6% over the five-year assessment period, whereas in the reshaping-group patients this increased from 32% to 66.9%.

The inter-observer agreement with regards to panel assessment was consistently high in each of the annual assessments (κ = 0.982, k = 1.00, k = 0.966, k = 1.00, k = 1.00; p < 0.0001 in all instances). BRA demonstrated a significant correlation to BIS on annual assessments (Spearman rank correlation coefficient and corresponding p-values annually: 0.248, p = 0.007; 0.364, p = 0.011; 0.409, p = 0.0001; 0.228, p = 0.021; 0.341, p = 0.0006).

## Discussion

This single-centre randomised controlled trial of 233 patients demonstrates that, as compared to BCS alone, simple oncoplastic closure of defects offers superior and durable cosmetic outcomes over a follow-up period of 5 years. The choice of oncoplastic technique will of course depend on the EEV, as well as the EEV/BV ratio. For the purposes of homogeneity, patients with an EEV/BV ratio of <20% were recruited in this study. Previous evidence shows that patient satisfaction after oncoplastic closure declines beyond an EEV/BV threshold of 20% [15]. Patients who required wide local excision of ≥20% of breast volume were considered for other oncoplastic procedures such as therapeutic mammoplasty or flap surgery, and were not recruited to this study.

Given that BCS is currently the standard of surgical care for breast cancer [1] and that approximately 13 of patients experience unsatisfactory cosmetic outcomes [2] with potentially deleterious effects to their wellbeing, it is encouraging to see that cosmesis can be improved with simple reproducible surgical techniques. The surgeons in our unit acquired the balanced experience to avoid extensive dissection and mobilisation, as this can increase the risk of fat necrosis and complications. In general, our recommendation is to keep the mobilisation of breast tissue and dissection to a minimum, whilst aiming to cover the chest wall muscle and repair the defect as much as possible.

Randomised controlled trial evidence is lacking in the field of cosmetic outcomes after breast cancer surgery. In their systematic review of oncoplastic BCS, *Haloua* et al. [1] analysed eleven non-randomised studies and found that cosmetic outcomes were adequately described in only a minority of studies (which collectively included 411 patients). Cosmetic assessment methods were typically non-uniform and non-validated. In this context, 84–89% of patients were deemed to have good cosmetic outcomes [1]. Patient satisfaction was reported in only one study, where 94% of 162 patients who underwent oncoplastic breast conserving surgery were either “satisfied” or “very satisfied” with their cosmetic outcome [15]. The systematic review authors concluded that the oncoplastic breast surgery evidence base is drawn from methodologically flawed and underpowered studies [1]. In a recent Cochrane review performed by *Nanda* et al. [9], oncoplastic closure was found to have similar or superior cosmetic results v standard BCS, but the authors noted that the evidence base is uncertain and prone to bias. Meta-analysis of the available non-randomised studies was not possible [9].

In the current study, randomisation, assessor blinding, and a combination of cosmetic assessment methods were used in order to maximise validity and generalisability. The combination of objective and subjective methods is supported by other investigators, as well as by the European Organisation for Research and Treatment of Cancer (EORTC) manual for clinical research in breast cancer (2005) [16]. Symmetry is arguably the most important feature of the global cosmetic picture and was objectively assessed using the well-described BRA tool. This objective measure of symmetry was in fact found to correlate (albeit weakly) to the subjective and patient-centred BIS. Combining BRA and BIS with the Harvard scale [13], which is considered one of the most widespread assessment scales in BCS [17], further increases the generalisability of our findings. Experienced observers from a high-volume breast cancer unit were involved in the cosmetic assessments and, as previously demonstrated by other groups [18], the inter-observer agreement in this study was consistently strong throughout the follow-up period.

In keeping with previous evidence [19], we found that the majority of patients were satisfied with the cosmetic outcomes in the reshaping group and were deemed to have a “good” or “excellent” cosmetic outcome by panel assessment. Similarly, the cosmetic outcomes of our control group assessed by clinician panel were in keeping with previous long-term (12 years) Danish evidence, where 50% (102/205) patients were deemed to have either “good” or “excellent” outcomes [20]. The corresponding figures in our study was 40–45% although this was assessed at 5 years postoperatively.

The main strengths of this study are its randomised controlled design, as well as its setting within a high-volume breast unit. Furthermore, this study analyses a patient group which is homogeneous in terms of the surgical techniques used. The procedure performed for the reshaping group is simple, quick (relative to more extensive oncoplastic techniques) and reproducible.

The limitations of this study include its single-centre setting, which may limit the generalisability of findings to a certain extent, as well as the fact that the assessment panel was composed of two individuals rather than the minimum five recommended by EORTC [16]. Unfortunately, arranging for five assessors on each occasion was not logistically possible in our unit.

In conclusion, patients with early primary breast cancer which requires excision of <20% of breast volume can benefit from simple oncoplastic closure of the defect after wide local excision in terms of long-term objective and subjective cosmetic outcomes. In this context, simple oncoplastic closure should be offered as standard treatment, assuming no compromise on oncological outcomes.

## Ethical approval

This study received ethical approval from the East of England – Norfolk Research Ethics Committee (Reference No.: 06/Q0101/34).

Mean breast retraction values are similar at baseline, and significantly lower (i.e. better) in the reshaping v the control group (p<0.0001) from year 1 onwards.

## Funding

Breast Surgery Unit Fund, 10.13039/501100009489Norfolk & Norwich University Hospital.

## Previous presentations

Findings from this study were presented at the European Society of Surgical Oncology (ESSO) 2020 Virtual Meeting in October 2020.

## Declaration of competing interest

The authors declare that they have no conflict of interest.
