# Supplementary material for: Simple oncoplastic breast defect closure improves long-term cosmetic outcome of breast conserving surgery for breast cancer: A randomised controlled trial
Source: Breast. 2022 Jul 18;65:104–9. doi: 10.1016/j.breast.2022.07.001 (PMC9356147; doi:10.1016/j.breast.2022.07.001)
Supplement: Multimedia component 2 [file mmc2.doc]

**
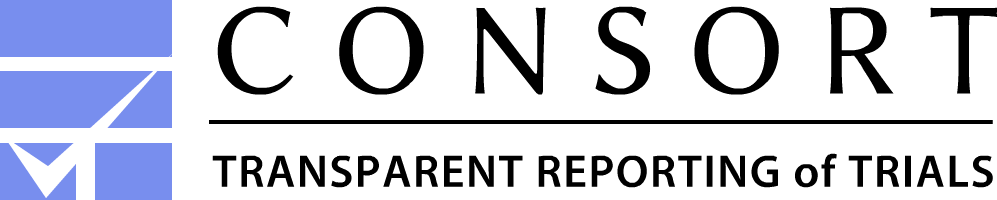
**

**CONSORT 2010 Flow Diagram**

**Allocation**

**Analysis**

**Follow-Up**

**Enrollment**

Assessed for eligibility (n=260)

Excluded (n=14)

♦  Declined to participate (n=14)

Analysed (n=109)
♦ Excluded from analysis (lost to follow-up) (n=11)

Lost to follow-up (give reasons) (n=11)

Allocated to control group (n=120)

♦ Received allocated intervention (n=120)

Lost to follow-up (give reasons) (n=2)

Allocated to intervention group (n=126)

♦ Received allocated intervention (n=126)

Analysed (n=124)
♦ Excluded from analysis (lost to follow-up) (n=2)

Randomized (n=246)
